# Healthcare professionals’ perceptions of managing psychological needs in palliative and end-of-life care: an All-Ireland mixed-methods survey

**DOI:** 10.1186/s12904-026-02262-2

**Published:** 2026-07-29

**Authors:** Niall Galligan, Barry Quinn, Rory Cousins, Deirdre Murphy, Brid McCarthy, Nipuna Thamanam, John Wonnacott, Mary Rabbitte, Fiona Somers, Lisa Graham-Wisener

**Affiliations:** 1 Our Lady’s Hospice and Care Services, Harold’s Cross, Dublin, Ireland; 2 School of Nursing and Midwifery, Queen’s University Belfast, Belfast, BT9 7BL UK; 3 Trinity College Dublin, College Green, Dublin, Ireland; 4 University College Dublin, Belfield, Dublin, Ireland; 5 Northern Health and Social Care Trust, Antrim Hospital Bush Road, Antrim, BT41 2RL UK; 6 All Ireland Institute of Hospice and Palliative Care, Harold’s Cross Rd, Harold’s Cross, Dublin, Ireland; 7 Voices4Care, All Ireland Institute of Hospice and Palliative Care, Harold’s Cross Rd, Harold’s Cross, Dublin, Ireland; 8 School of Psychology, Queen’s University Belfast, Belfast, BT7 1NN UK

**Keywords:** Psychological, Palliative care, End-of-life care, Hospice, Life-limiting, Healthcare professional, Ireland, Survey

## Abstract

**Background:**

Despite recognition in clinical guidelines and quality standards, integration of psychological care into palliative and end-of-life care settings remains inconsistent across and within health systems. Structural, workforce and training challenges persist as key barriers. This study provides the first investigation of healthcare professionals’ perspectives on the assessment and support of psychological needs within adult palliative and end-of-life care across the island of Ireland.

**Methods:**

A convergent mixed-methods design, with online survey data from 152 healthcare professionals working in palliative and end-of-life care settings across the island of Ireland. Quantitative data were analysed descriptively and qualitative data using reflexive thematic analysis.

**Results:**

The findings indicate that although psychological support is perceived as foundational to palliative and end-of-life care, this does not appear to be prioritised in relation to workforce development and service delivery. Healthcare professionals report a lack of training opportunities, issues with accessing specialist psychology resource, and time constraints that hinder their ability to assess and address psychological needs effectively.

**Conclusions:**

There is an urgent need for investment in psychological care across adult palliative and end-of-life care settings on the island of Ireland. Integration of specialist psychology resource and enhancing training pathways for the broader workforce are essential to implementing effective stepped care models that ensure psychological needs are met.

**Supplementary Information:**

The online version contains supplementary material available at https://doi.org/10.1186/s12904-026-02262-2.

## Background

The psychological dimensions of serious illness have long been recognised as integral to high-quality palliative and end-of-life care [1]. Dame Cicely Saunders’ concept of ‘total pain’, which highlights the interplay of psychological suffering with physical, social and spiritual suffering [2], continues to shape how healthcare professionals understand and respond to the needs of patients and informal carers. Psychological care of individuals with life-limiting illness involves both the amelioration of distress of all types and levels of severity, as well as the promotion of psychological wellbeing through a focus on meaning-making, coping resources and building resilience [3–5]. Psychological distress exists on a continuum, from normal emotional responses to clinically significant presentations of anxiety, depression and other complex mental health conditions [6]. Individuals with life-limiting illness experience increased risk of severe forms of psychological distress [7], which present a central and pervasive challenge to broader symptom management and the overall goals of palliative care. Clinically significant distress is associated with increased symptom burden [8], a lack of acceptance of prognostic information [9], and a reduced health-related quality of life [10]. There is also an evidenced interdependency between patient-carer dyads in relation to psychological distress [11]. Notably, psychological distress is a recognised contributor to the multidimensional complexity that often underpins referrals to specialist palliative care services [12].

For these reasons, the need for high-quality psychological care, as part of holistic care, is consistently referenced in international clinical guidelines and quality standards for both generalist and specialist palliative care [3–5, 13, 14]. Guidelines often recommend delivery of a stepped-care model of psychological support [3–6, 14], in which individuals begin with the least resource-intensive intervention (universal support) and are ‘stepped up’ to targeted, specialist, or highly specialised care as needed, based on the complexity of need. Stepped-care models aim to improve the efficiency and equity of care delivery by providing timely access to support while protecting specialist mental health resources. However, there is potential for suboptimal outcomes if stepped-care models are not implemented effectively, particularly in relation to assessment, monitoring, and timely escalation. Within this model, the majority of psychological care is provided by non-specialists who provide universal or targeted support. Dependent on the complexity of need, healthcare professionals with further training (e.g. counsellors) would provide specialist support. Mental health professionals, largely psychologists, then represent a highly specialist resource, providing advanced assessment and complex intervention, as well as supporting the wider multidisciplinary team through consultation, supervision and training [15, 16].

Despite recognition in clinical guidelines and quality standards, integration of psychological care into palliative and end-of-life care settings remains inconsistent across and within health systems, and is often limited by structural, workforce and training challenges. Drawing on the UK as an example, recent evidence indicates that more than a quarter of nurses and allied health professionals working in hospices in England have no level two (*targeted support*) training, and that healthcare professionals feel unable to screen and assess patients for referral to specialist services as these are perceived as significantly lacking [17]. Similarly, a wider UK survey of adult hospices [18] indicated that only 16% of organisations rated their psychological service as wholly adequate. The majority of services reported they can access specialist professionals, but many rely on external referrals [18].

While international and UK evidence provides key insights into workforce challenges in palliative and end-of-life care provision more broadly, its applicability to the island of Ireland cannot be assumed. The island of Ireland is composed of Northern Ireland and the Republic of Ireland, with separate healthcare systems yet increasing cross-border collaboration, particularly in border regions and facilitated through all-island professional networks. Cross-border service delivery introduces further complexity in professional roles. Relevant clinical guidelines recommend a stepped-care model, with healthcare professionals across generalist and specialist palliative care with the appropriate competencies to attend to a continuum of complex need [3, 5]. In comparison to the international context, Northern Ireland and the Republic of Ireland operate under devolved or distinct healthcare systems, with differing funding models and commissioning arrangements which shape the delivery of psychological care. Moreover, Northern Ireland is frequently underrepresented or excluded from UK-wide health services research, limiting the relevance of existing evidence to this context.

While the same concerns are echoed anecdotally in palliative and end-of-life care settings on the island of Ireland, there remains a lack of evidence-based understanding of how psychological care is integrated and delivered. Delivering on psychological care in palliative and end-of-life settings is a shared challenge for the island of Ireland, with public consultation exercises by the All-Ireland Institute of Hospice and Palliative Care identifying the unmet emotional and psychological needs of individuals with serious illness, informal carers, and families as a key priority [19, 20]. The importance of improving psychological support is also reflected in recent strategic commitments across both jurisdictions, including the Northern Ireland and Republic of Ireland Cancer Strategies and national palliative care policies [4, 21, 22]. Examining the perspectives of healthcare professionals is particularly important, as they are responsible for identifying and addressing psychological needs, yet their experiences, challenges, and training requirements remain underexplored across the island.

Given the growing policy focus and cross-border collaboration, it is timely to examine the provision of psychological care from a workforce perspective. Currently, there is a lack of empirical evidence on how psychological needs are identified and supported within adult palliative and end-of-life care settings across the island of Ireland, or on the competencies and training needs of those delivering this care. This study addressed that gap, with the aim of informing workforce development and supporting the implementation of policy commitments in both jurisdictions.

### Aim

To investigate healthcare professionals’ understanding, practices, and challenges related to the assessment and support of psychological needs within adult palliative and end-of-life care settings across the island of Ireland, and to identify training and resource requirements to enhance psychological care delivery.

### Objectives

I. To explore healthcare professionals’ understanding of the role of psychological care within palliative and end-of-life care (*qualitative*)
II. To explore healthcare professionals’ involvement in and perceived competence with assessing and supporting the psychological needs of patients and family carers in palliative and end-of-life care settings (*quantitative*)
III. To identify how healthcare professionals identify psychological needs in palliative and end-of-life care settings, and how they support psychological needs once identified (*quantitative*)
IV. To examine the challenges healthcare professionals face in delivering psychological care in palliative and end-of-life care settings, and the training and resources needed to improve practice (*quantitative & qualitative*)

## Methods

### Study design

A convergent mixed-methods design, with data collected via a single online survey. The survey was predominantly quantitative, with a subset of qualitative items. The mixed-methods approach was justified by the study’s aim and objectives, which require both breadth and depth of understanding [23]. Quantitative data provided insight into the prevalence and patterns of current practices, confidence levels, and resource gaps. Qualitative data allowed participants to elaborate on their experiences, challenges, and perceived training needs, adding contextual richness and interpretive depth to the numeric findings. The online survey method was chosen as it enables recruitment of a geographically dispersed sample and may encourage greater disclosure on potentially sensitive topics (e.g. confidence and perceived limitations in delivering psychological care) [24]. This study was informed by a critical realist perspective, assuming a reality shaped by but not reducible to individual experience. This aligns with a mixed-methods approach, combining measurable patterns with contextual meaning.

This study is reported according to the CHERRIES Checklist for Reporting Results of Internet E-Surveys [25] and the Mixed Methods Reporting in Rehabilitation & Health Sciences Checklist (MMR-RHS, [26]).

### Population and setting

The setting for the study was the island of Ireland, including Northern Ireland with a population circa 1.9 million [27] and the Republic of Ireland with a population circa 5.3 million [28]. Palliative care is a recognised specialism in both Northern Ireland and the Republic of Ireland, with well-developed hospital, hospice and home care services [29]. The overall palliative care service provision is considered ‘established’ in Northern Ireland and ‘integrated’ in the Republic of Ireland [29], with integrated viewed as a more advanced stage of service development denoting routine collaboration across health and social care sectors.

The eligibility criteria for the study included adults (> 18 years) currently working in a professional capacity in any adult palliative and end-of-life setting on the island of Ireland. For this study, palliative and end-of-life care settings were defined to include both specialist and generalist services providing care to adults with life-limiting illnesses, across hospice, hospital and community. Interdisciplinary palliative and end-of-life care teams on the island of Ireland include doctors, nurses, social and pastoral care workers, allied health professionals and in a minority of cases, psychologists.

Participants were recruited through convenience sampling, with an open-survey link shared via the All-Ireland Hospice and Palliative Care (AIIHPC) professional network mailing list. Convenience sampling may have resulted in self-selectin bias; however, this approach was appropriate for the study’s exploratory aim of mapping current practice across a geographically dispersed workforce. AIIHPC is a collaborative of 27 hospices, health and social care organisations, charities and universities on the island of Ireland. AIIHPC members were encouraged to complete and also share the survey link to colleagues who may wish to complete the survey. A total of two reminder emails were circulated. The AIIHPC mailing list has *n* = 999 recipients, encompassing a range of individuals including healthcare professionals, researchers and policymakers.

### Survey design and implementation

The survey was developed by the co-authors, interdisciplinary researchers, clinicians and patient and public involvement representatives who represent the working group of the AIIHPC Psychological, Social and Spiritual Special Interest Group. The survey aimed to explore current practice and need relating to psychological and spiritual care in palliative and end-of-life care settings. These domains were prioritised as key areas of focus by the working group, reflecting their importance in practice and their close interconnection in supporting patient well-being. The spiritual needs data is the basis of another paper. A review of the literature informed the domains of interest, and in particular several previous workforce related surveys on psychological and spiritual care [17, 30, 31]. Refinement of the survey (focus on clarity, relevance and burden) was informed by feedback from external experts, and PPI representatives, with final refinement undertaken after piloting. Survey items were presented across five sections. In the first two sections, demographic and professional background information was collected. The remaining three sections captured both quantitative and qualitative data on current practice, perceived competence and training needs regarding psychological and social care. See Supplementary Material 1.

The short (< 15 min), 27-item online survey was hosted on Microsoft Forms online platform. Participants read a participant information sheet (including the purpose of the study, length of time of the survey, data storage procedures and who is managing the research) and provided explicit informed consent before completing the survey. Survey items were presented to participants across 8 pages (range 4–10 items per page) and in a standardized order. Respondents were able to review and change their answers. Responses were required to all items, with the exception of qualitative items. Due to the platform used, IP addresses and cookies were not collected and so there is the possible risk of multiple entries (though inspection of data did not indicate any). Responses were collected between August 2024 and October 2024. Median completion time was approximately 14 min. No financial incentive was offered for completion. No personal data were collected.

### Ethics

Research ethics approval was provided by the Queen’s University Belfast Medical and Health Sciences Faculty Research Ethics Committee (MHLS 24_128) on 20th August 2024.

### Patient and public involvement

This research study was identified as a priority through discussions with the AIIHPC Psychological, Social and Spiritual Special Interest Group, which represents interdisciplinary researchers, clinicians and patient and public involvement representatives from across the island of Ireland. A PPI representative co-author from Voices4Care has been involved from the early stages of designing the research. Having previously cared for two members of their immediate family over a five-year period, both of whom had cancer, they considered this topic was important to research as they witnessed firsthand the connection and impact between the physical and psychological states of their family members at various stages of their illnesses. Every step of the study was designed and implemented in conversation with those who had lived experience, adding to the richness of the study.

### Data analysis

#### Quantitative data

Data were exported from Microsoft Forms and analysed using the Statistical Package for Social Science for Windows, Version 25 (SPSS Inc., Chicago, IL, USA). Only the data pertaining to psychological needs are the basis for this manuscript. The spiritual needs data is the basis of another paper. As responses were forced, there is no missing data. Likert scale data were treated as ordinal. Absolute numbers and percentages were reported.

As no inferential statistical analyses were planned, the sample size was determined based on the need for clear, interpretable proportions, rather than statistical power. With a final sample of 152 participants, we are able to report key proportions (e.g., the percentage who feel competent) with a margin of error of approximately ± 8% at the 95% confidence level. A response rate could not be calculated because the survey was distributed via an open professional network and further disseminated through participant sharing, meaning the total number of individuals who received the survey invitation is unknown.

#### Qualitative data

There were two qualitative items within the survey focused on psychological needs- ‘Could you describe in your own words the role of psychological care in palliative care?’ and ‘What further training would help you to identify and support patients’ and family/carer’s psychological care needs?’. There were 125 free-text responses to the first question, and 118 free-text responses to the second question. Free-text responses were each between one sentence and several paragraphs in length.

Different analytic approaches were selected to align with the aims of each qualitative item: reflexive thematic analysis [32, 33] enabled in-depth conceptual exploration of how psychological care is understood, while conventional content analysis [34] facilitated pragmatic categorisation of training needs to inform workforce development.

Reflexive thematic analysis was used to inductively analyse the data from the first question, using both semantic and latent coding. The data were analysed by a male assistant psychologist (RC), working clinically in a hospice environment and so representing an ‘insider’ perspective. The six-phase process of reflexive thematic analysis [32, 33] was undertaken. This involved becoming familiar with the data, generating initial codes, developing and reviewing themes, refining and naming them, and producing a final analytic narrative that captured meaningful patterns across the dataset.

Free text responses to the second question - ‘What further training would help you to identify and support patients’ and family/carer’s psychological care needs?’ - were analysed using a conventional content analysis approach to identify recurring themes and patterns in healthcare professionals’ views. The data were analysed by a clinical neuropsychologist (NG), working clinically in a hospice environment and so representing an ‘insider’ perspective. Non-informative entries (e.g., “not sure) were removed and remaining responses were then systematically reviewed and categorised into thematic groups based on key terms and concepts. The resulting category framework was reviewed by a second researcher, a research psychologist (LGW), to support analytic clarity and transparency.

#### Data integration

Consistent with a convergent mixed-methods design, quantitative and qualitative data were analysed independently using their respective analytic approaches and integrated during the interpretation phase. Integration involved considering how findings from the two data strands related to one another in addressing the overall study aim and objectives.

The extent of integration varied according to the focus of each objective. Some objectives were primarily addressed through a single methodological strand, with qualitative findings informing understanding of healthcare professionals’ conceptualisation of psychological care and quantitative findings describing reported practices, confidence, and patterns of psychological care provision. Where both quantitative and qualitative data addressed related aspects of the research questions, findings were compared narratively during interpretation to identify areas of convergence, complementarity, and divergence.

Integration was most evident in relation to challenges in delivering psychological care and the training and resources required to improve practice. Quantitative findings indicated the extent to which barriers, resource limitations, and training needs were reported across participants, while qualitative findings provided contextual insight into the nature of these challenges and the types of support healthcare professionals considered important. The combined interpretation of findings informed the overall conclusions and recommendations regarding psychological care provision in palliative and end-of-life care settings.

## Results

### Participant characteristics

A total of 152 responses were obtained and analysed, with respondents working in the following settings: Hospice (38.41%, 58/151); Community (29.14%, 44/151); Hospital (16.56%, 25/151); across multiple settings (13.25%, 20/151) and Other (2.65%, 4/151). Participants held diverse roles in palliative and end-of-life care with nurses making up the majority of respondents, but with representation from an array of disciplines. The majority of respondents had over 10 years of experience in palliative and end of life care (Table 1).

**Table 1:** Participant Characteristics

Variable	Category	No. of Participants	%	Total (*N*)
Role	Nurse	73	48.03%	152
	Social Worker	18	11.84%	
	Chaplains / Pastoral Care worker	14	9.21%	
	Occupational Therapist	10	6.58%	
	Medical Doctor	8	5.26%	
	Physiotherapist	5	3.29%	
	Dietitian	5	3.29%	
	Pharmacist	5	3.29%	
	Psychologist	3	1.97%	
	Other / not specified	11	7.24%	
Age	18–24	1	0.66%	152
	25–34	13	8.55%	
	35–44	34	22.37%	
	45–54	55	36.18%	
	55–64	47	30.92%	
	65–74	2	1.32%	
Length of time supporting people in Palliative and end-of-life care settings	Less than a year	8	5.26%	152
	1–5 years	31	20.39%	
	6–10 years	25	16.45%	
	> 10 years	88	57.89%	

The majority of the sample were women (82.2%, 125/152). Most participants identified as Christian (70.30%, 107/152), followed by spiritual but not religious (18.4%, 28/152). Participants varied in age, ranging from 18 years of age upwards, with the highest proportion of respondents falling within the 45–54 age bracket.

### Healthcare professionals’ understanding of the role of psychological care within palliative and end-of-life care

Four themes were constructed in the exploration of healthcare professionals’ understanding of the role of psychological care within palliative and end-of-life care.

#### Theme 1: positioning psychological care as central to holistic palliative care

Participants consistently constructed psychological care as a central component to palliative and end-of-life care. It was described not as an optional component but as a central pillar embedded within the broader framework of holistic care. Psychological care was considered foundational in addressing the complex emotional, existential, and relational needs of patients and their families.

“*Psychological care is the cornerstone of palliative care: giving the patient or family - child or adult - the opportunity to share their worries and fears in a safe and supportive environment.”* [Participant 11, Woman, Social Worker, Hospice setting].

*“Overlapping with spiritual care… probably one of the most important aspects of palliative care provision.”* [Participant 14, Woman, Nurse, Community setting].

These accounts highlight the blurring of disciplinary boundaries, where psychological care is embedded in everyday relational work within all roles.

#### Theme 2: creating a therapeutic space for both patients and their family

Participants emphasised the significance of psychological care in providing a safe and therapeutic space where patients and their families can openly express emotional and existential concerns. This environment, grounded in therapeutic presence, a non-judgemental approach, trust and compassion, was seen as a key therapeutic intervention in itself.

*“Having a protected space to explore feelings freely with a skilled practitioner… is so important.”* [Participant 10, Woman, Nurse, Hospice setting].

*“Psychological care offers the patient and family an opportunity to express their fears and worries… and resolve practical and emotional matters important to them before their death.”* [Participant 25, Woman, Social Worker, Hospice setting].

Psychological care was described as involving dedicated time and space to work through practical concerns impacting emotional wellbeing (e.g., adaptation to illness-related limitations) as well as more abstract or distal matters (e.g., historical events). This theme also captures the need for a specialist skill set to hold this space effectively. Psychological care was therefore constructed as not simply being present, but a skilled practice.

#### Theme 3: psychological care as both relief and resilience building

Participants described psychological care as a process that helps patients identify and understand their psychological distress while simultaneously enhancing resilience. While reliving suffering remained a core concern, equal importance was placed on supporting people to mobilise all available coping resources to navigate the complexity of serious illness.

*“To explore the impact of the illness journey… to assess what support may be needed in alleviating any psychological distress…” *[Participant 44, Woman, Social Worker, Community setting].

*“Supporting patients to explore all aspects of their disease and how this impacts on their emotional, psychological, cognitive, behavioural and social wellbeing as well as reflecting on the impact of pre-morbid mental health conditions.”* [Participant 81, Woman, Occupational Therapist, Hospice setting].

Specific emphasis is placed on gaining a nuanced understanding of each person’s unique illness journey, mental health history, and family dynamics, in order to mobilise all available support systems and coping resources. Psychological care was therefore framed as tailored and responsive, not a one-size-fits all, with emphasis on the importance of building bespoke care plans for the person and their family that optimises awareness of, and engagement with, multidimensional supports.

“*Assess the strengths and levels of resilience within a family… while considering additional strategies if appropriate.”* [Participant 6, Man, Social Worker, Community setting].

#### Theme 4: leveraging past experiences to navigate present and future challenges

Participants described a role for psychological care in bringing an appreciation for the patient’s life narrative and the strengths they have developed over time, as part of a person-centred ethos. In the process of doing so, psychological care was described as supporting individuals to reflect on past coping mechanisms, and consider what is useful or relevant in navigating current and future challenges, thus enhancing self-efficacy and meaning-making while preparing for future challenges.

“*Building with the patient/family from their past experience*,* to live as well as possible in the now and sometimes plan for the future.”* [Participant 4, Woman, Nurse, Hospital, hospice and community settings].

“*Exploring what mechanisms they have used to help them through difficult times and who they may seek out for support.”* [Participant 84, Man, Doctor, Hospital, hospice and community settings].

The centrality of loss as a theme in palliative and end of life care, be it past, present or future losses, makes psychological care a practical and appropriate focus. These accounts emphasise that psychological care was not viewed as separate from the person’s broader life narrative, but rather as a bridge between the past and present which supports emotional adjustment.

### Healthcare professionals’ involvement in and perceived competence with assessing and supporting the psychological needs of patients and family carers

Psychological needs are widely recognised by respondents but seldom assessed or addressed systematically. Most participants (80.26%, 122/152) reported ‘frequent’ or ‘very frequent’ involvement in psychological support. Only a minority (7.2%, 11/152) reported that they were never involved in assessing and supporting patient or family/carer’s psychological needs.

**Fig. 1:**
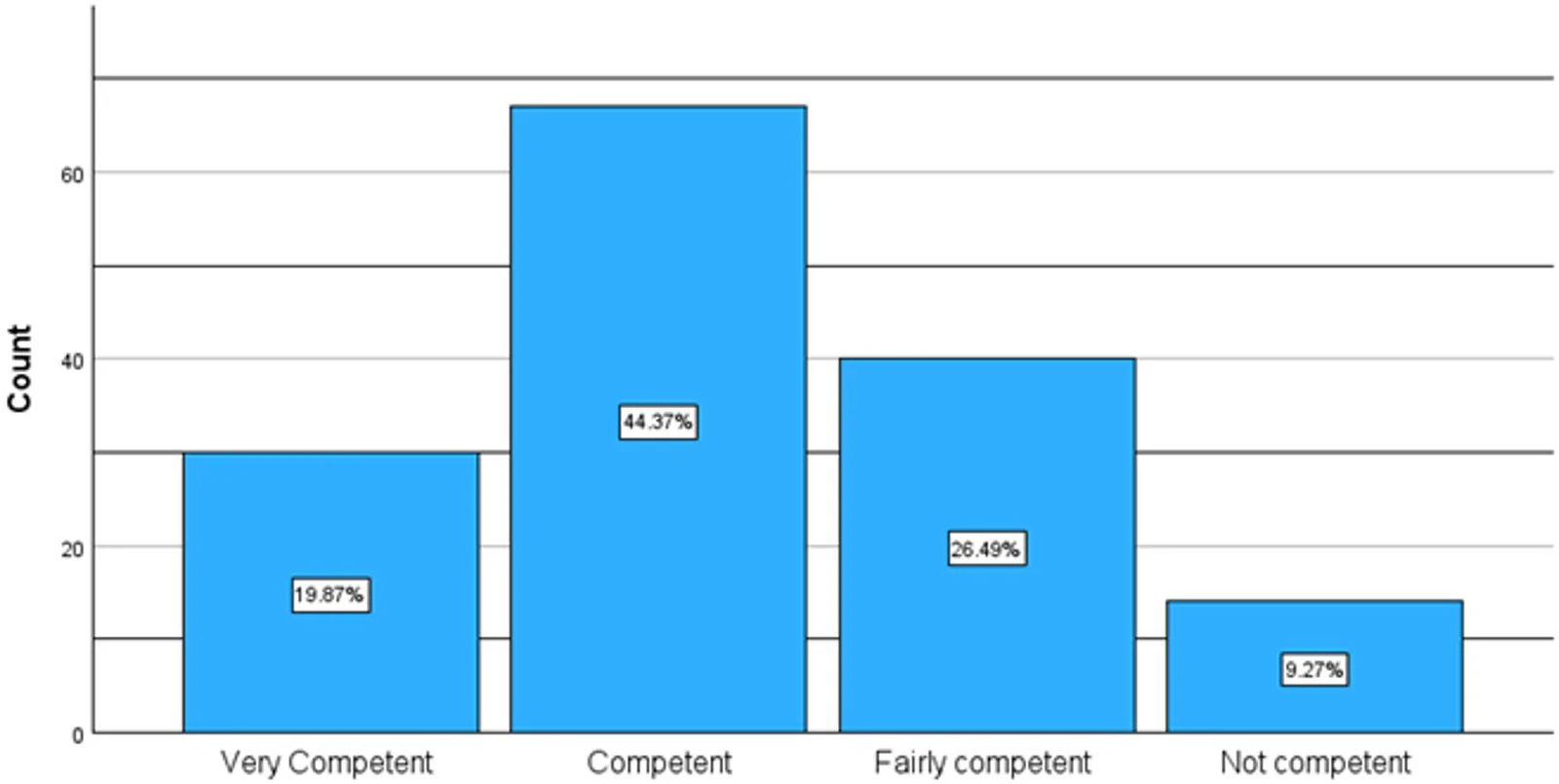
How would you rate your competence in supporting patients' and family/carers' psychological needs?

A majority (63.82%, 97/152) described themselves as either ‘very competent’ or ‘competent’ in supporting patients’ and family / carers’ psychological needs (Fig. 1).

**Fig. 2:**
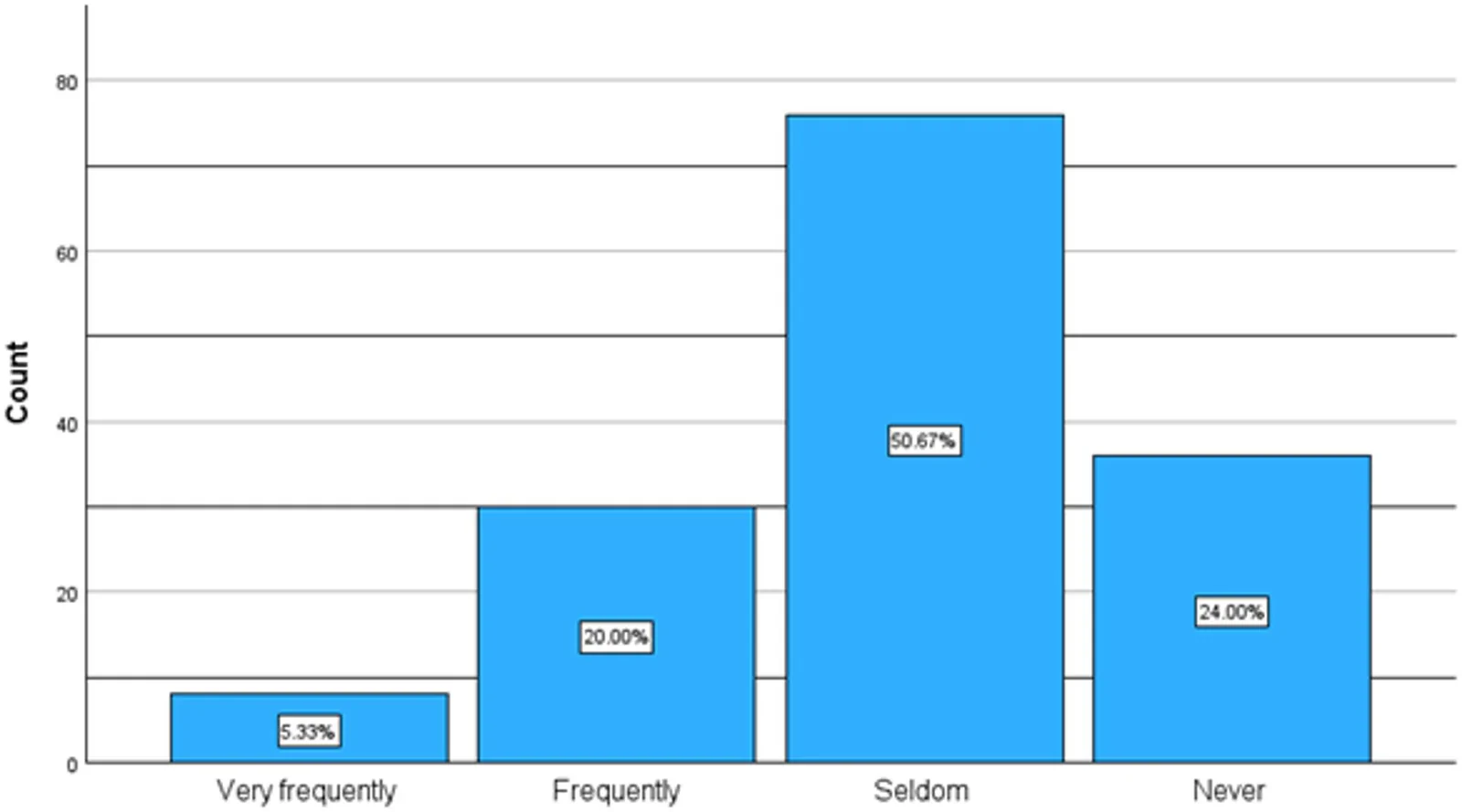
How often have you received training focused on psychological care?

In contrast, the majority (73.68%, 112/152) of respondents described having ‘seldom’ or ‘never’ received training focused on psychological care (Fig. 2).

### How healthcare professionals identify and support psychological needs in palliative and end-of-life care settings

**Fig. 3:**
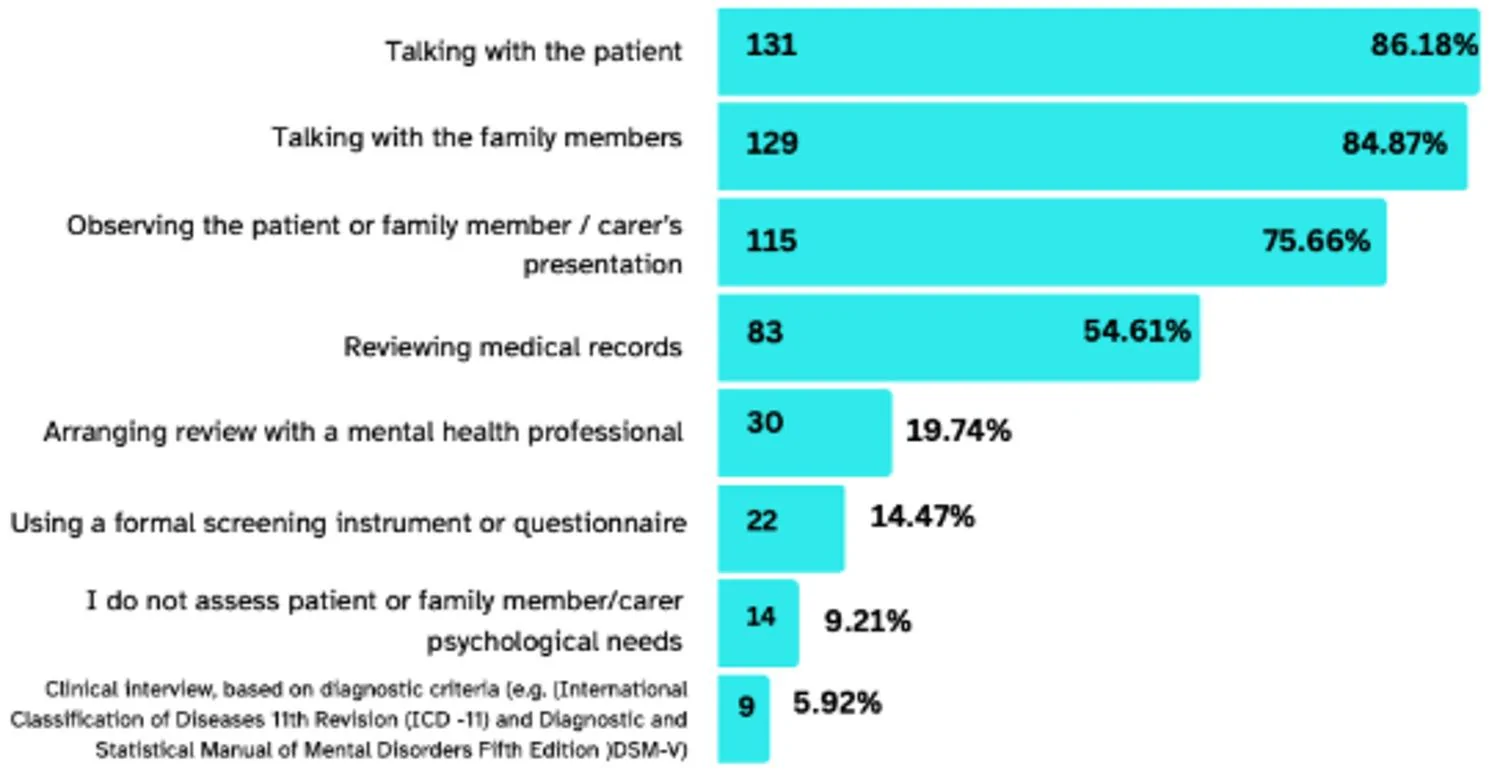
How do you assess patients' and family / carers' psychological needs?

In relation to how healthcare professionals identify psychological needs, talking with patients (86.18%, 131/152), talking with family (84.87%, 129/152), patient observation (75.66%, 115/152) and reviewing medical records (54.61%, 83/152) were the most common methods of assessment selected. Use of formal tools (e.g., screening questionnaires or clinical interviews based on DSM-5- TR or ICD-11) were selected less often, suggesting reliance on subjective or informal assessment methods (Fig. 3).

**Fig. 4:**
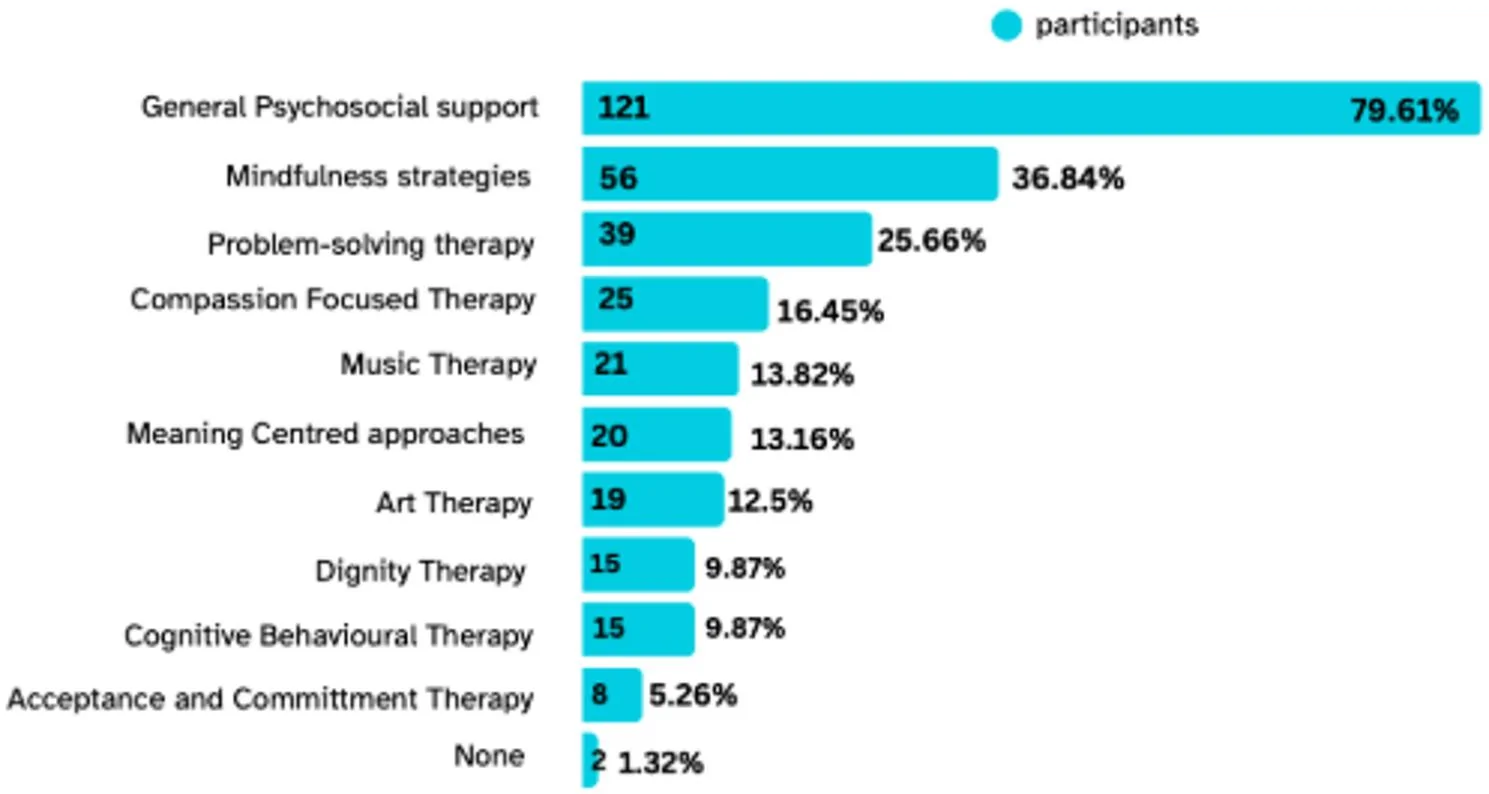
What (nonpharmacological) approaches do you use to support patients' or family / carers' psychological needs?

In terms of supporting psychological needs, once identified, the most commonly selected support approaches included general psychosocial support (79.61%, 121/152), mindfulness (36.84%, 56/152), and problem-solving approaches (25.66%, 39/152). Other evidence-based psychological approaches were less commonly selected, and included Compassion Focused Therapy (16.45%, 25/152), Meaning Centred approaches, (13.16%, 20/152), Dignity Therapy (9.87%, 15/152), and Acceptance and Commitment Therapy (5.26%, 8/152). This indicates a preference for low-intensity, non-structured interventions in most settings, though availability of training or trained professionals likely influences modality use (Fig. 4).

**Fig. 5:**
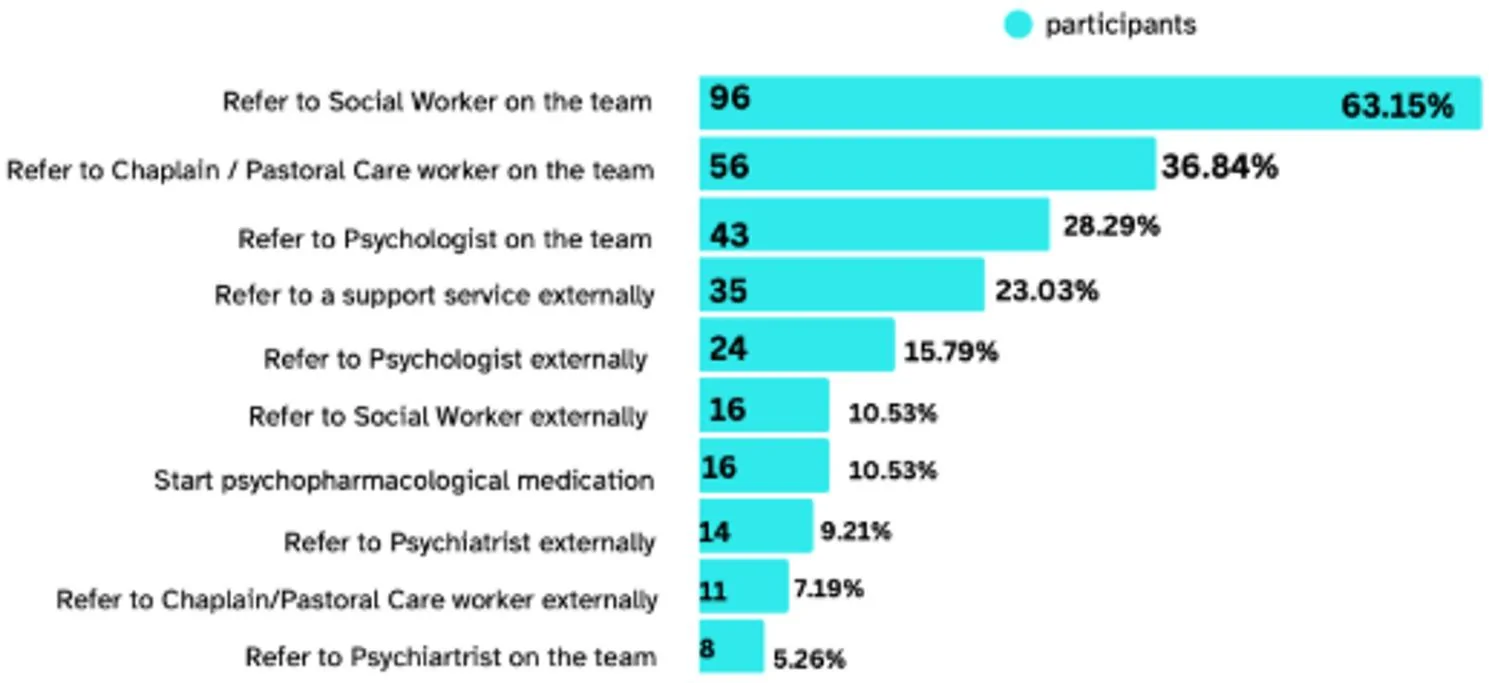
If you identify any type of psychological needs in a patient or family / carer, what do you do next?

The ways in which respondents and services address psychological needs often depend on staff skill mix and resource availability. Onward referrals to address psychological needs were most commonly selected by respondents to be to social workers (63.15%, 96/152) and pastoral care services (36.84%, 56/152), while 28.29% (43/152) of respondents reported referring to a psychologist on their team. Initiation of psychopharmacological medication was reported by 10.53% (16/152) of respondents (Fig. 5).

### Challenges healthcare professionals face in delivering psychological care in palliative and end-of-life care settings, and resources needed to improve practice

**Fig. 6:**
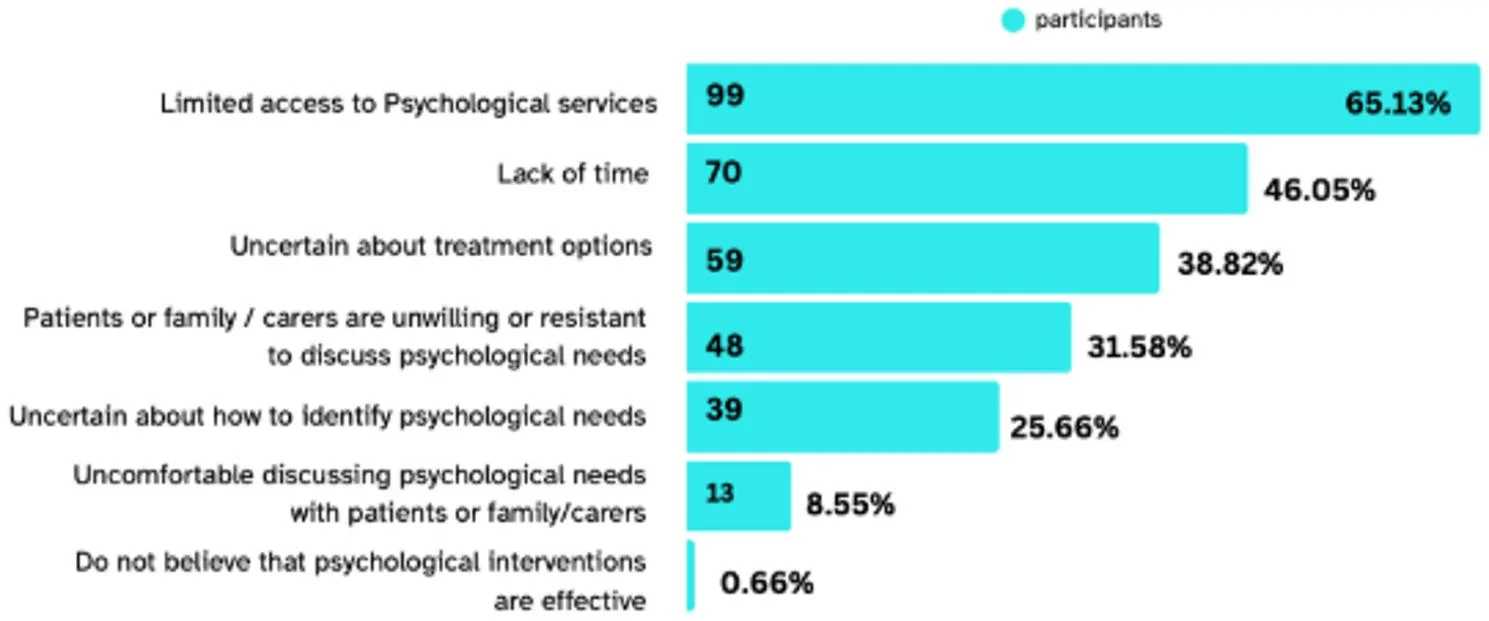
What are the main challenges for delivery of psychological care in palliative care?

A number of systemic barriers were identified which impede the effective provision of psychological care. Top barriers endorsed included limited access to specialist psychological services (65.13%, 99/152), lack of time (46.05%, 70/152), and uncertainty about treatment options (38.82%, 59/152). Around a quarter (25.66%, 39/152) were unsure how to identify psychological needs. These findings suggest structural and educational gaps in psychological service integration (Fig. 6).

When asked what resources could help the respondent’s organisation or team to address psychological needs, from pre-defined items participants highlighted the following;

- Staffing: 69.4% (106/152) endorsed the need for additional staff with expertise in psychological care/mental health.
- Training: 66.45% (101/152) endorsed the need for more training in how to address and manage psychological needs.
- Time: 51.32% (78/152) endorsed the need for more time to spend with patients and families.
- Technology: 28.95% (44/152) endorsed greater use of relevant digital tools and resources.

Participants were then asked an open-ended question, on what future training would help them to identify and support patient and family member psychological needs. Participant responses centred around the following three overarching categories:

- Assessment and identification of psychological needs,
- Referral pathways and service signposting,
- Communication skills,

The most commonly identified area of training need was in relation to assessment of service-users’ psychological needs. Specifically, training on the use of evidence-based screening tools for psychological distress in day-to-day practice was the most frequently cited training need. Training in basic psychological intervention skills was also sought. This included introductory training on mindfulness, Cognitive Behavioural Therapy, meaning centered approaches and Compassion Focused Therapy, where relevant therapeutic techniques could then be integrated into day-to-day clinical encounters as part of a generalist approach to supporting service-users’ mental health.

“*Assessment skills - use of distress thermometer and how to assess clearly the nature of the need and know who best to refer to*” [Participant 12, Woman,, Nurse, Hospice setting].

“*Basic level psychological support intervention training guided by specialist psychological support professionals*” [Participant 80, Woman, Nurse, Hospital setting].

A related training need identified was guidance at a local level on who and where to refer individuals identified as experiencing elevated psychological distress to. Training was seen as a helpful way to communicate appropriate and available channels to access mental health professionals and services.

“*Training on awareness of where who and how to refer patients and family to services for their psychological care needs*” [Participant 88, Woman, Pharmacist, Hospital, hospice, and community settings].

“*Awareness about support services and how to access them*” [Participant 44, Woman, Nurse, Hospice setting].

The final category identified concerned training on how best to approach sensitive or challenging conversations such as when staff encounter negative patient/family dynamics or unhelpful coping responses (e.g., denial). Participants reported an openness to both online (e.g., webinars and e-courses) and in-person training formats (e.g., workshops) and identified a need for both brief skills building events (e.g., 1-day workshops) as well as greater access to post-graduate training as part of their continuous professional development (e.g., post-graduate diploma up to master’s level training).

“*More training on difficult conversations*,* and services available*” [Participant 129, Woman, Occupational Therapist, Community setting].

“*Online Training/ Video using Local (Island of Ireland) scenarios*” [Participant 123, Woman, Nurse, Hospice setting].

## Discussion

The current study offers the first insight into current perspectives on addressing psychological needs within specialist and generalist palliative and end-of-life care settings across the island of Ireland. In doing so, it adds to the small body of international research exploring healthcare professionals’ understanding, practices, and challenges related to the assessment and support of psychological needs (e.g. [17, 18]). Responses were gathered from a sample of largely very experienced healthcare professions, representing a range of disciplines, and who work in a broad spectrum of settings, including hospices, hospitals, and community-based services. Key findings indicate that although psychological support is considered by healthcare professionals as central to palliative and end-of-life care, a perceived mismatch is observed between the importance attributed to psychological care and the resources available to support its delivery. Healthcare professionals report a lack of training opportunities, time constraints and issues with accessing specialist mental health resource, which they describe as an impediment to assessing and addressing psychological needs effectively.

Beyond these specific findings, several overarching inferences emerged from respondents’ accounts. First, a perceived discrepancy was noted between the importance of psychological care and the resources available for its delivery within palliative and end-of-life care services. Second, relatively high levels of self-reported competence coexisted with low levels of reported participation in formal training in psychological care; the majority of respondents described having ‘seldom’ or ‘never’ received training focused on psychological care. While limited formal training does not necessarily equate to limited competence, this finding raises questions about how competence in psychological care is developed, maintained, and assessed across services. Third, respondents’ accounts suggest that the delivery of psychological care is not an isolated skill or responsibility; it is influenced by interacting factors where potential areas for improvement are noted across individual (training, experience and knowledge development), team (role delineation and collaborative practice) and system (service design and staffing to enhance access to specialist psychologist input) levels.

A notable finding from this study is the coexistence of healthcare professionals’ self-reported competence in addressing psychological needs and their infrequent reported participation in formal training, alongside an expressed need for further education. Given the experience profile of the sample, participants may have developed relevant knowledge and skills through clinical practice, informal mentorship, interdisciplinary working, supervision, postgraduate education, or continuing professional development that they did not classify as formal psychological training. Conversely, self-reported competence does not provide an objective measure of clinical knowledge and skill nor does it establish whether practice is consistently aligned with clinical guidance. While many respondents felt competent in providing psychological care, few reported using validated screening tools or structured clinical interviews as recommended by clinical guidelines (e.g., [3]). This suggests that perceptions of competence may not always reflect guideline-concordant or best practice, potentially due to gaps in awareness, as noted in a recent UK study [17]. Further research using more detailed measures of training exposure and practice would be needed to understand this relationship. There were also a sizeable proportion of respondents who reported being unsure about treatment options. Furthermore, respondents identified communication as a critical area requiring further training and support. This underscores a need for education and training, focusing on psychological assessment, triage, and referral pathways. While university curricula may have evolved to place greater emphasis on psychological care, the current sample largely comprised experienced clinicians, highlighting the ongoing need for continuing professional development to ensure psychological competencies are maintained and updated in line with current best practice.

These findings also highlight the importance of delineating roles and competencies within multidisciplinary teams to avoid over-reliance on a generalist model for psychological care. While all team members have a role in recognising and responding to psychological distress, further evidenced by the majority of respondents indicating they are frequently involved in psychological support, complex presentations often require input from professionals with specialised psychological training [35]. Without clear pathways for escalation to specialist care, there is a risk that psychological needs remain insufficiently addressed, despite best intentions [36]. While the study did not directly evaluate the quality or outcomes of the psychological support currently provided, the reported limited use of formalised assessment methods and evidence-based psychological therapies, such as cognitive behavioural approaches, Compassion-Focused Therapy (CFT) or meaning-centred approaches, represents a potential focus for service development. Generalist psychosocial support is an important component of palliative care and may be sufficient for many patients. However, some patients with more complex or persistent difficulties, may benefit from assessment or intervention by professionals with specialist psychological expertise (e.g., to address issues such as historical trauma, severe anxiety, harmful coping or behavioural patterns, or unresolved existential distress). Clear referral and escalation pathways may therefore help multidisciplinary teams match the level of support to the nature and complexity of patients’ needs. For example, unrecognised trauma-related distress may complicate care and may sometimes be expressed through physical or somatic symptoms, consistent with the multidimensional concept of “total pain” as described in palliative literature [37]. Moreover, coordinated trauma-informed communication and care planning at a team level, can help avoid inadvertent re-traumatisation, and support therapeutic engagement [38, 39].

Importantly, limited access to specialist psychological services was the most frequently endorsed barrier to the effective provision of psychological care, endorsed by the majority of respondents. Only, a minority of respondents in the current study reported having direct access to a psychologist, which is also an issue reported in both hospice and non-hospice settings across the UK [17, 40]. The new Adult Palliative Care Policy for the Republic of Ireland [4] hopes to improve access with recommended psychology staffing levels per population and specialist palliative care inpatient unit size, and its implementation may help address some of the access difficulties identified by respondents. Although the survey did not directly assess whether stepped-care models were in place or how effectively they were implemented, the reported difficulties concerning training, access to specialist psychological expertise, and referral pathways may indicate challenges in delivering some components of stepped care. Evidence from service contexts internationally suggests that psychologists working within palliative care teams support a stepped care model by contributing to team consultation, staff confidence, specialist assessment, and timely intervention [3, 4, 39]. They may also support the development of generalist staff competencies through training, supervision and informal consultation.

Despite these challenges, the importance of psychological care is well-acknowledged by respondents, who consistently highlighted it as a key and interconnected component of holistic, person-centred, end-of-life care. Psychological distress may be shaped by, and may in turn influence, physical symptoms, functional changes, family and social circumstances, financial pressures, spiritual concerns, and existential uncertainty. The creation of a therapeutic environment that promotes psychological safety and non-judgement is essential for patients to articulate and process emotional concerns that might otherwise be expressed indirectly or remain unrecognised. In many cases, these stressors may manifest indirectly as physical pain, fatigue, or disengagement from medical care. Providing patients with a psychologically safe space facilitates the transition from vague emotional discomfort to clearer, shared understanding - an essential step for therapeutic healing and growth in the face of life limiting illness. Psychological care at the end of life is not solely about managing distress but also focuses on facilitating adjustment and enhancing emotional wellbeing. As respondents noted, it provides a platform to build on patients’ existing strengths, resilience, and coping repertoires. This strength-based approach enables patients to draw from past experiences and internal resources to navigate current and future challenges. Meaning-making at the end of life has been shown to significantly reduce existential distress and improve emotional well-being [41].

This study has various strengths, including the recruitment of a large sample of healthcare professionals from across palliative care settings on the island of Ireland. In terms of limitations, the majority of responses came from nursing professionals, though this is likely representative of the regional palliative care workforce. The study did not independently assess clinical competence, patient outcomes, or adherence to clinical guidance. It also did not directly examine the presence, implementation, or effectiveness of stepped-care models. The findings are based on healthcare professionals’ self-reports and therefore represent their perceptions of competence, practice, and barriers to service provision. Consequently, conclusions regarding these issues should be regarded as hypotheses for further investigation rather than direct findings of the present study. It must also be noted that the majority of participants reported a Christian religious affiliation. This demographic homogeneity may limit the generalisability of findings across other cultural or religious traditions, such as Islamic or Buddhist contexts, where the conceptualisation of psychological and spiritual needs can differ due to varying cultural frameworks. This research also focused exclusively on healthcare professional views and needs in terms of provision of psychological care within a palliative and end-of-life care context. Future research could focus on exploring the perspectives of service-users and family members on the experience of psychological care in this context. Finally, given the clear interest and need for further staff education and training in this area, there is an opportunity for future research on the design, implementation and effectiveness of such interventions within this context.

## Conclusion

Palliative care requires an integrated, multidisciplinary approach that that attends to the interacting physical, psychological, social, and spiritual dimensions of patient and family experience. Within this context, the present study provides a focused examination of psychological care as one component of holistic practice. Respondents regarded psychological care as an important component of palliative care but reported challenges relating to training opportunities, time, role clarity, referral pathways, and access to specialist psychological support. Addressing these challenges through structured training, clearer role delineation, robust clinical governance, and improved access to specialist input has the potential to enhance outcomes for both patients and staff. Integration of specialist psychological expertise within multidisciplinary palliative care teams also aligns with recent adult palliative care policy in Ireland [4] which is in the process of implementation. Further research, with regards to psychological care in palliative and end-of-life care contexts, should examine patient and family perspectives, objectively assess professional knowledge, skills and service model implementation, and evaluate the impact of changes in training and specialist provision of psychological care on overall care processes and outcomes.

## Supplementary Information

Supplementary Material 1.

Supplementary Material 2.

## Data Availability

The datasets used and/or analysed during the current study are available from the corresponding author on reasonable request.
