# Supplementary material for: Healthcare professionals’ perceptions of managing psychological needs in palliative and end-of-life care: an All-Ireland mixed-methods survey
Source: BMC Palliat Care. 2026 Jul 29;25:324. doi: 10.1186/s12904-026-02262-2 (PMC13700532; doi:10.1186/s12904-026-02262-2)
Supplement: Supplementary file 1 [file 12904_2026_2262_MOESM1_ESM.pdf]

Appendix 1: Survey

All Ireland Institute of Hospice and Palliative Care Network

Exploring Spiritual and Psychological Care Survey

Part One: Demographics

Please tick one of the following that best describes your age?

18-24

25-34

35-44

45-54

55-64

65-74

75 and older

Please tick one of the following that best describes your gender?

Woman

Man

Non-binary

Queer-gender

Other (please state)

Please tick one of the following that best describes your pronoun?

She/Her

He/Him

They/Them

Other (please state)

Please tick one of the following that best describes your religion/spirituality?

Christianity

Buddhism

Hinduism

Humanism

Islam

Judaism

Sikhism

Neither spiritual or religious

Spiritual but not religious

Other (please state)

Part Two: Introductory questions

Please tick one of the following that best describes your role in palliative and end of life care

Chaplain

Doctor

Nurse

Occupational Therapist

Pastoral Care Worker

Physiotherapist

Psychiatrist

Psychologist

Psychotherapist

Social worker

Other (please state)

How long have you been engaged in supporting people receiving palliative and end-of-life care? (please tick one)

Less than 1 year

1-5 years

6-10 years

>10 years

What kind of service do you work in? (please tick those most relevant to you)

Hospital

Hospice

Community

Other (please state)

Part Three: Spiritual Care

How often are you involved in assessing and supporting patients' and family/carers' spiritual needs. (please tick one)

(very frequently/frequently/seldom /never)

How would you rate your competence in supporting patients' and family/carers' spiritual needs? (please tick one)

(very competent/ competent / fairly competent /not competent)

Could you describe in your own words the role of spiritual care support in palliative care? (free text)

How do you assess patients' and family/carers' spiritual needs? (please tick all that apply)

- Using a screening instrument or questionnaire (please specify what instrument(s)
- Through a patient's medical/nursing records
- Talking with the patient
- Talking with the family
- Observing the patient's presentation
- Arranging a review with a Chaplain / Pastoral Care worker
- I do not assess patient's spiritual needs

If you identify any type of spiritual needs in a patient or family/carer, what do you do next? (Please tick all that apply)

- Offer them my support
- Refer to a Chaplain / Pastoral Care worker

    - on the team
    - externally
- Refer to Psychologist or counsellor
    - on the team
    - externally
- Refer to a support service (externally)
- Other (Please specify)

What interventions and approaches do you use to support patients' or family/carers' spiritual needs? (please tick all that apply)

- Provide an opportunity to talk
- Offer to bring them to a prayer room
- Offer to bring them to a quiet place
- Offer to bring them to the garden
- Get advice from the chaplain/pastoral care worker
- Suggest or offer mindfulness
- Suggest or offer meditation
- Suggest or offer prayer
- Compassion Focused Therapy
- Dignity Therapy
- Music therapy
- Art therapy
- Other (please state)

What are the main challenges for the delivery of spiritual care in palliative care? (please tick all that apply)

- Limited access to spiritual care services
- Lack of time
- Uncertain about how to identify spiritual needs
- Uncertain about support options
- Do not believe that spiritual interventions are effective
- Uncomfortable discussing spiritual needs with patients/family/carers
- Patients/family/carers are unwilling or resistant to discuss spiritual needs
- Other (please specify)

What resources could help you/your organisation/team address spiritual needs more effectively? (please tick all that apply)

- Additional staff with expertise in spiritual care
- More training in how to identify and address spiritual needs
- More time to spend with patients
- Use of relevant technology (e.g. mobile apps, internet-based resources, etc.)
- Other (please specify)

How often have you received training focused on spiritual care? (please tick one)

(very frequently/frequently//seldom/ never)

Part 4: Psychological Care

How often are you involved in assessing and supporting patient and family/carers' psychological needs? (please tick one)

(*very frequently/frequently/seldom /never)*

How would you rate your competence in supporting patients' and family/carers' psychological needs? (please tick one)

(very competent/ competent / fairly competent /not competent)

Could you describe in your own words the role of psychological care in palliative care? (free text)

How do you assess patients' and family/carers' psychological needs? (please tick all that apply)

- Using a formal screening instrument or questionnaire (Please specify what instrument(s)
- Clinical interview, based on diagnostic criteria (e.g. ICD-11, DSM-V)
- Reviewing medical records
- Talking with the patient
- Talking to the family members
- Observing the patient or family member/carers' presentation
- Arranging review with a mental health professional
- I do not assess patient or family member/carer psychological needs

If you identify any type of psychological needs in a patient or family/carer, what do you do next? (please tick all that apply)

Offer them my support

- Refer to Social Worker
  - on the team
  - externally
- Refer to Psychologist
  - on the team
  - externally
- Refer to Psychiatrist on the team

- on the team
- externally
- Refer to Chaplain / Pastoral Care worker
  - on the team
  - externally
- Start psychopharmacological medication
- Refer to a support service (externally)
- Other (Please specify)

What nonpharmacological approaches do you use to support patients' or family/carers' psychological needs? (please tick all that apply)

- General psychosocial support
- Problem-solving therapy
- Mindfulness strategies
- Meaning Centred Approaches
- Cognitive Behavioral Therapy
- Acceptance and Commitment Therapy
- Compassion Focused Therapy
- Dignity Therapy
- Music therapy
- Art therapy
- Other (please state)

What are the main challenges for delivery of psychological care in palliative care? (please tick all that apply)

- *Limited access to psychological services*
- *Lack of time*
- *Uncertain about how to identify psychological needs*
- *Uncertain about treatment options*
- *Do not believe that psychological interventions are effective*
- *Uncomfortable discussing psychological needs with patients or family/carers*
- *Patients or family/carers are unwilling or resistant to discuss psychological needs*
- *Other (please state)*

What resources could help you/your organisation/team address psychological needs more effectively among your patients and their family/carers? (please tick all that apply)

- Additional staff with expertise in psychological care / mental health
- More training in how to identify and treat psychological needs
- More time to spend with patients and/or family/carers
- Use of relevant technology (e.g. mobile apps, internet-based resources, etc.)
- Other (Please specify – free text)

How often have you received training focused on psychological care? Please tick one)

(very frequently/frequently//seldom/ never)

Section 5 Training

What further training would help you to identify and support patients' and family/carer's spiritual care needs? (free text)

What further training would help you to identify and support patients' and family/carer's psychological care needs? (free text)
